# PolicyMap

**DOI:** 10.5195/jmla.2020.858

**Published:** 2020-01-01

**Authors:** David Farris

**Affiliations:** Research Medical Library, University of Texas MD Anderson Cancer Center, Houston, TX, dpfarris@mdanderson.org

## INTRODUCTION

PolicyMap is an online geographic information system (GIS) mapping tool that aggregates many types of data for users to create maps and reports for research, grant applications, health policy, market surveys, and other applications. According to the Reinvestment Fund, PolicyMap originally started in 2009 as a small project by 2 employees “to help government agencies, nonprofit organizations and housing professionals easily understand and incorporate geographic data into their decision-making processes” [1]. Since then, it has grown to incorporate over 37,000 data indicators from more than 150 public and private agencies to power the platform. Although the data are amassed from many different sources, they are cleaned and normalized to reduce redundancy and maintain integrity. In addition to federal, state, and local governments, PolicyMap is used by educational institutions, foundations, nonprofit organizations, and health care systems to help make impactful decisions.

## MAJOR FEATURES

**Figure 1:**
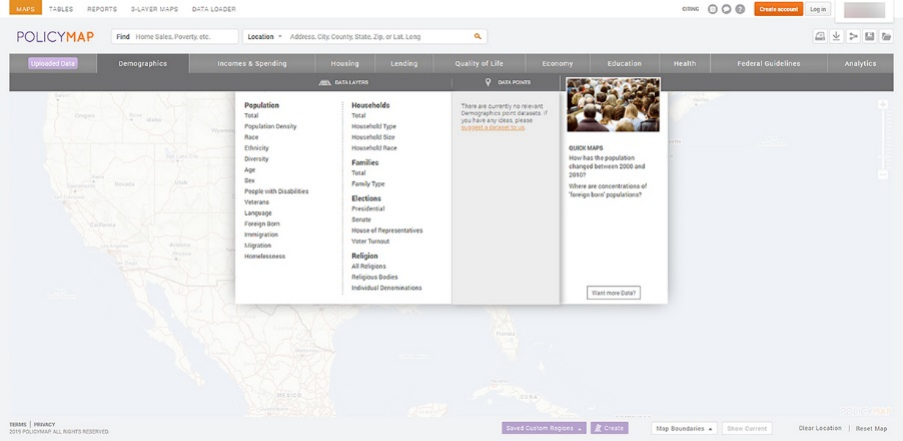
Data categories and data layers and data points for the demographics category

Currently, PolicyMap encompasses ten broad categories of data: demographics, income and spending, housing, lending, quality of life, economy, education, health, federal guidelines, and analytics. Data in each category are then divided into two groups: data layers and data points (Figure 1). Data layers are displayed as thematic or heat maps, and the data are categorized into more granular levels within each data layer. For example, the health category offers six primary categories of data layers: vital statistics, health status, risk factors, costs and insurance, access to medical care, and food access. The vital statistics data layer includes three options, and each option may provide access to even more specific data. Data points are displayed as icons for point-level datasets overlaid on thematic maps, and they are divided into two broad categories: health facilities, and food and grocery retail access. As with data layers, each of these categories may offer more specific types of data.

PolicyMap features five main components: Maps, Tables, Reports, 3-Layer Maps, and Data Loader. Each component offers users the ability to save, print, download, and share maps, tables, reports, and other user-generated content; however, some of these functions are not available in Open Map, the freely available public edition. In addition to these five functions, PolicyMap has announced that it will release a new version in January 2020 with new and enhanced features, such as multilayer maps, additional zoom levels, and three-dimensional (3D) mapping.

The Map function feature offers users the ability to display data at the address, zip code, county, state, or longitude and latitude level or select from predetermined geographies such as census tract, congressional district, and school district. Once the location is selected, users can explore different categories of data; for example, demographics, quality of life, education, health, and federal guidelines. Data can be displayed in layers or as points on the map. Users are able to zoom in and out or pan anywhere on the map to explore additional data.

The Tables function allows users to display data for one or more locations and compare data across multiple geographies in tablature format rather than as a map. As with the Maps function, once they select a location, users can load data from the data menus or the data search bar. Users can explore data at a more granular level by selecting Values, Lists, or Rankings. The Values option allows users to see and download all the data values for all the geographies that make up a selected area. The Lists option displays all the addresses from a point data set that are contained in a selected geography; for example, the number of libraries located in the state of Texas. The rankings option allows users to see where that location ranks among other locations in the same data set and the top and bottom ten locations in the data set.

PolicyMap allows users to generate four kinds of reports for either predefined locations, such as state or census tract, or custom locations specified by the user: community, rental housing, Home Mortgage Disclosure Act (HMDA), and community health. The community report includes information related to a location’s population, income, age, and additional demographic data, and the rental housing report includes rental information according to size and cost. The HDMA report provides information related to home mortgages and lending activity, and the community health report provides a summary of health care information for a location. All of these reports, with the exception of the community health report, are available to non-subscribers.

The 3-Layer Maps function is a powerful tool for exploring neighborhood data, and it allows users to find neighborhoods that match from one to three types of data to display on a map. For example, a user might wish to see which neighborhoods in a specific geographical location are eligible for the New Markets Tax Credit (NMTC) program and then see the median household income and the estimated percentage of people under the age of eighteen living in those neighborhoods. Each of these criteria is added to the map as a layer, and the subsequently shaded portion of the map corresponds to the matching data. PolicyMap also allows users to edit the data shown on each level, such as a specific time period or income range. The 3-Layer Maps feature is only available to subscribers.

Subscribers can use the Data Loader function to upload their own datasets to PolicyMap and then overlay PolicyMap data to create and share unique address-level datasets. This is an advanced function: it involves multiple steps to create the custom map and requires the user’s comma separated value (CSV) file to be formatted in a specific way to upload properly into PolicyMap. Advanced GIS users will most likely benefit the most from this feature due to its complexity.

## USABILITY AND ACCESSIBILITY

PolicyMap is designed to be easy to use with little or no previous experience; however, less intrepid users may find the platform a bit intimidating at first because it is loaded with an abundance of disparate types of data and many features for mapping it. There are several brief tutorials and videos on the PolicyMap YouTube channel and a comprehensive primer to help researchers learn about each of the features. PolicyMap maintains the *MapChats Blog*, the official outlet for sharing information about how users can get the most out of the platform. PolicyMap also complies with Section 508 Accessibility Standards for Electronic and Information Technology (EIT) and is compatible with assistive technology.

## PRICING LEVELS AND PURCHASE OPTIONS

PolicyMap offers a tiered subscription model based on the size and type of institution, organization, agency, or company and the number of options desired. In addition to enterprise licenses, PolicyMap offers a university license that grants unlimited concurrent access to faculty and students. Individuals can register for a free account with access to the mapping feature and public data sets or buy a subscription to use standard or premium options. PolicyMap also offers data licenses for those who wish to embed maps and data on a website or to use with business intelligence tools. Subscribers can use PolicyMap’s Data application programming interface (API) to supply data directly to custom maps or tables or request flat files to integrate data into custom analytics.

## CONCLUSION

PolicyMap is a powerful tool for using data to study location-specific socioeconomic issues and inform policy makers at the federal, state, and local levels. Subscriptions and free accounts with limited features are available as well as resources for using them. Due to its exhaustive and expertly curated library of data indicators, especially those “related to health and social determinants of health that is potentially relevant to public and community health researchers, practitioners, and librarians” [2], PolicyMap is a worthwhile addition to other electronic resources offered by academic and health sciences libraries.

## 

**David Farris, AHIP,** dpfarris@mdanderson.org, https://orcid.org/0000-0002-9224-9270, Research Medical Library, University of Texas MD Anderson Cancer Center, Houston, TX
